# Precision Cardio-Oncology and Nuclear Imaging: Current Applications, Molecular Innovations, and Future Trajectories

**DOI:** 10.3390/cancers18101625

**Published:** 2026-05-18

**Authors:** Biruk Demisse Ayalew, Muhammad Areeb Ul Haq, Talha Farooq, Moosa Mubarika, Muhammad Umar, Urvah Shafique, Abdullah Rehman, Hassan H. Eladl, Abad Ahmad Toor, Eman Fatima, Temesgen Mamo Sharew, Mirza Mohammad Ali Baig, David N. Smith, Daniel Addison

**Affiliations:** 1 Department of Internal Medicine, St. Paul’s Hospital Millennium Medical College, Addis Ababa 1000, Ethiopia; 2 Department of Internal Medicine, Allama Iqbal Medical College, Lahore 54550, Pakistan; 3 Jinnah Sindh Medical University, Karachi 75510, Pakistan; 4 Nishtar Medical University, Multan 66000, Pakistan; 5 Khairpur Medical College, Khairpur Mir’s 66020, Pakistan; 6 Medical Research Group of Egypt (MRGE), Negida Academy, Arlington, MA 11511, USA; 7 Faculty of Medicine, Ains Shams University, Cairo 11566, Egypt; 8 Islamic International Medical College, Riphah International University, Rawalpindi 46000, Pakistan; 9 School of Medicine, Yale University, New Haven, CT 06520, USA; 10 Cardio-Oncology Program, UT Southwestern Medical Center, Dallas, TX 75390, USA; 11 Division of Cardiovascular Medicine, UT Southwestern Medical Center, Dallas, TX 75390, USA

**Keywords:** cardio-oncology, nuclear imaging, cardiotoxicity, cancer therapy, early detection, precision medicine

## Abstract

Cardiovascular toxicity has emerged as a major determinant of long-term outcomes in cancer survivors as advances in oncologic therapies continue to improve survival. Conventional cardiac surveillance strategies predominantly rely on functional and structural changes, often identifying myocardial injury after clinically significant damage has occurred. The aim of this narrative review is to critically evaluate the role of nuclear imaging in advancing precision cardio-oncology by enabling earlier, mechanism-based detection and characterization of cancer therapy-related cardiotoxicity. We summarize current clinical applications of PET- and SPECT-based imaging, examine molecular and tracer-level innovations, and discuss emerging hybrid imaging and analytic approaches relevant to individualized cardiovascular risk stratification. Current literature indicates that nuclear imaging provides unique insights into myocardial perfusion, metabolism, inflammation, and microvascular dysfunction, facilitating detection of subclinical injury across diverse anticancer therapies, including anthracyclines, targeted agents, and immune checkpoint inhibitors. By integrating molecular imaging with conventional modalities, nuclear techniques support more personalized surveillance and management strategies. This narrative review highlights nuclear imaging as an emerging complementary modality within precision cardio-oncology supporting earlier detection and risk stratification, and outlines future directions required to optimize its clinical integration and impact on cardiovascular outcomes across the cancer care continuum.

## Simple Summary

Cancer treatments are helping people live longer, but they can also quietly damage the heart. Current methods usually detect heart problems only after significant injury has already occurred. This research explores how advanced imaging techniques, particularly nuclear imaging, can identify early and hidden heart damage before symptoms appear. We aim to explain how these technologies work, what new developments are emerging, and how they can improve patient care. By detecting problems earlier, doctors may be able to adjust cancer treatment and protect the heart more effectively. This could lead to more personalized care, better long-term outcomes for cancer survivors, and a shift in how researchers and clinicians approach the balance between treating cancer and preserving heart health.

## 1. Introduction

### 1.1. The Evolving Interface Between Cancer Therapy and Cardiovascular Disease

Over the past two decades, substantial advances in anticancer therapy, including targeted molecular agents, immunomodulators, and cellular therapies, have markedly improved survival across a broad spectrum of malignancies [1,2]. However, these therapeutic gains have been accompanied by increasing recognition of cardiovascular complications, such as left ventricular dysfunction, heart failure, arrhythmias, ischemic events, and vascular toxicities [3,4]. As cancer survivorship continues to expand, cardiovascular disease has emerged as a leading cause of morbidity and mortality among cancer survivors [5]. This convergence of oncology and cardiology has driven the development of cardio-oncology, a specialized discipline dedicated to the identification, prevention, and management of cancer therapy-related cardiovascular toxicity [6].

### 1.2. Spectrum of Cancer Therapy-Related Cardiovascular Toxicities

Cancer therapies are associated with a broad spectrum of cardiovascular toxicities that vary according to mechanism of action and duration of exposure. These include left ventricular systolic dysfunction and heart failure, particularly with anthracyclines and HER2-targeted therapies; immune checkpoint inhibitor-associated myocarditis; ischemia and vasospasm related to fluoropyrimidines; and microvascular dysfunction induced by anti-angiogenic agents. In addition, arrhythmias and QT interval prolongation are frequently observed with targeted therapies. Radiotherapy contributes to long-term complications, including accelerated coronary artery disease, valvular heart disease, pericardial disease, and conduction abnormalities. The heterogeneity of these toxicities underscores the need for imaging strategies capable of capturing structural, functional, and molecular alterations across different stages of disease progression. This review builds upon recent EANM, EACVI, and AHA scientific statements by specifically focusing on nuclear imaging’s evolving role within precision-guided cardio-oncology.

### 1.3. Limitations of Conventional Surveillance and the Need for Earlier Detection

Despite growing awareness of cardiotoxicity and the establishment of surveillance frameworks, significant challenges remain in identifying myocardial injury at a stage when intervention can meaningfully alter outcomes [1]. Clinical symptoms and physical examination, while fundamental, often reflect advanced myocardial involvement rather than early injury [4,7]. Echocardiography, particularly left ventricular ejection fraction (LVEF) and global longitudinal strain (GLS), has become the cornerstone of routine surveillance; however, these parameters may fail to capture subtle molecular or metabolic alterations preceding functional decline [7,8]. Electrocardiography remains valuable for arrhythmia detection and QT interval monitoring, especially in patients receiving tyrosine kinase inhibitors, yet its role in early myocardial injury detection is limited [6]. Circulating biomarkers such as cardiac troponins and natriuretic peptides (BNP/NT-proBNP) provide prognostic insight but lack sufficient specificity to consistently identify subclinical cardiotoxicity across diverse therapeutic exposures [9,10,11]. Collectively, these limitations underscore the need for more sensitive and mechanistically informative diagnostic approaches. Collectively, these limitations underscore the need for more sensitive and mechanistically informative diagnostic approaches.

### 1.4. Nuclear Imaging as a Molecular Lens in Precision Cardio-Oncology

Despite the use of these tools, conventional strategies often lack the specificity and sensitivity required to detect subtle or early cardiotoxic changes, thereby underscoring the need for more precise nuclear imaging techniques [12,13].

Within this context, nuclear imaging has emerged as a complementary modality capable of identifying molecular and functional patterns of cardiotoxic injury at an early clinical stage. In contrast to echocardiography and cardiac magnetic resonance (CMR), which primarily provide structural and functional assessments, nuclear imaging offers insights into cellular metabolism, myocardial perfusion, sympathetic innervation, and inflammatory activity. These parameters are particularly relevant for evaluating the heterogeneous cardiotoxic effects of cancer therapies such as anthracyclines, HER2-targeted agents, tyrosine kinase inhibitors, and immune checkpoint inhibitors, each of which induces myocardial dysfunction through distinct pathophysiological pathways. Nuclear techniques, including positron emission tomography (PET) and single-photon emission computed tomography (SPECT), facilitate early detection of therapy-related cardiac injury, enable assessment of treatment response, and enhance understanding of the underlying mechanisms of cardiotoxicity [12,13].

### 1.5. Rationale and Scope of This Review

Despite increasing interest in nuclear imaging within cardio-oncology, its clinical integration remains inconsistent, and evidence is dispersed across modalities, tracers, and disease contexts. Existing diagnostic strategies continue to prioritize functional decline rather than molecular disruption, limiting the realization of truly precision-guided cardio-oncology care. In this context, nuclear imaging represents a potential bridge between mechanistic understanding and individualized risk stratification.

**Figure 1:**
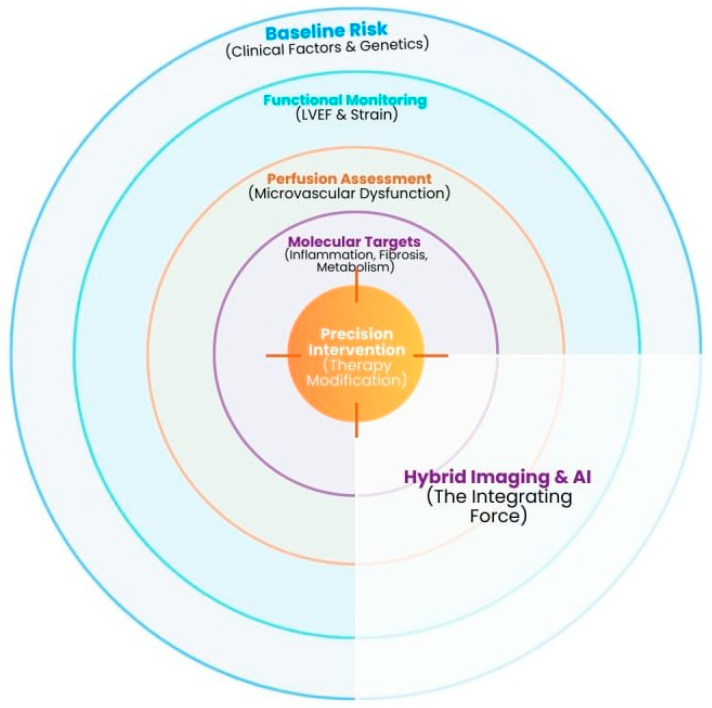
Flow chart showing the workflow of nuclear and multimodality imaging in cardio-oncology.

This narrative review synthesizes current clinical applications, molecular innovations, and emerging directions in nuclear imaging relevant to precision cardio-oncology. By integrating established modalities such as SPECT and ^18F-FDG PET with evolving tracer development and AI-enhanced analytical frameworks, this review aims to contextualize the role of nuclear imaging in refining early detection, improving risk stratification, and informing personalized cardiovascular management in cancer survivors. Importantly, this review positions nuclear imaging within a multimodality framework, emphasizing its complementary role alongside echocardiography, cardiac magnetic resonance, and computed tomography, rather than as a standalone replacement. (Figure 1).

## 2. Methods

### 2.1. Literature Search Strategy

This study was conducted as a narrative (non-systematic) review. A literature search was performed to identify studies examining the current applications, molecular innovations, and future directions of nuclear imaging within the field of precision cardio-oncology. Publications from database inception through August 2025 were considered to ensure comprehensive coverage of both foundational and contemporary evidence. The electronic databases PubMed, Embase, and Scopus were searched using combinations such as Medical Subject Headings (MeSH) and free-text keywords. Search terms included combinations of “Precision Cardio-Oncology,” “Nuclear Imaging,” “Molecular Imaging,” “Cardiotoxicity,” “PET/CT,” “SPECT,” “Immune Checkpoint Inhibitor Myocarditis,” and “Radiation-Induced Heart Disease.”

### 2.2. Eligibility Criteria

Studies were eligible for inclusion if they were published in English, peer-reviewed, and addressed at least one of the following domains: clinical applications of nuclear imaging in cardio-oncology, molecular or tracer-based innovations, or future trajectories relevant to precision cardiovascular care in oncology populations. The eligibility criteria for this narrative review included studies focusing on PET, SPECT, or hybrid imaging in cardio-oncology and target populations receiving potentially cardiotoxic therapies. While exclusion criteria consisted of case reports with *n* < 5 and studies without clear cardiac specific endpoints.

### 2.3. Study Selection Process

Given the narrative nature of this review, study selection followed a structured but non-systematic approach. After removal of duplicate records, titles and abstracts were independently screened by multiple reviewers using predefined inclusion and exclusion criteria. Full-text evaluation was subsequently performed for eligible articles. Additional relevant studies were identified through manual reference list screening. Any discrepancies in study selection were resolved through discussion with an additional reviewer to achieve consensus.

### 2.4. Narrative Synthesis

A narrative and thematic synthesis was conducted to evaluate the current applications of nuclear imaging in cardio-oncology, focusing on its role in early detection and monitoring. Novel tracers and hybrid molecular techniques were analyzed in the context of cost, accessibility, and protocol variability to provide an integrated overview of the role of nuclear imaging in precision cardio-oncology. Given the narrative design, the selection of studies may be subject to selection bias, and findings should be interpreted accordingly.

## 3. The Established Role of Nuclear Imaging in Cardio-Oncology

### 3.1. Foundational Applications: LVEF and Perfusion

Left ventricular ejection fraction (LVEF) is one of the most important metrics of the left ventricle (LV) systolic function, acting as an indirect indicator of the contractility of the myocardium. Although LVEF is an important parameter for identifying considerable cardiac dysfunction because of chemotherapy, most of the time it will only decline after considerable damage to the myocardium has already taken place [14]. Nuclear imaging may identify damage before LVEF decline occurs by identifying less severe conditions that could affect LVEF, including diminished myocardial blood flow and alterations in the metabolism of glucose and fatty acids [15].

Rubidium-82 (Rb-82) or Ammonia-13N (13N) PET imaging are two methods that can measure the integrity of the microcirculation, with the latter having the ability to provide quantitative measurements of myocardial perfusion as well. In this sense, nuclear imaging has a two-fold purpose: it can measure LVEF with great reliability for monitoring, while also identifying early signs of cardiotoxicity by assessing deficits in tissue metabolism and perfusion [16].

### 3.2. Multi-Gated Radionuclide Angiography (MUGA/ERNA)

Multi-gated Radionuclide Angiography (MUGA), also known as Equilibrium Radionuclide Angiography (ERNA), has historically been used as a valuable tool in cardio-oncology for reproducible assessment of LVEF across multiple evaluations [17]. It provided a reliable alternative for serial LVEF measurement in patients where echocardiography was limited by a restrictive acoustic window. By quantifying systolic function, MUGA enabled early detection of chemotherapy-induced cardiotoxicity in patients at elevated clinical risk [18,19]. In addition, assessment of phase function and analysis of LVEF alongside other markers, such as elevated approximate entropy, offered important prognostic information regarding the risk of developing cancer therapy-related cardiac dysfunction (CTRCD) [20].

With the development of more advanced imaging techniques, MUGA has largely been replaced. Today, 3D echocardiography (with or without contrast) and cardiac MRI are preferred for accurate serial assessment, volumetric analysis, and tissue characterization, as they are more effective and avoid the radiation exposure associated with MUGA [18]. As a result, MUGA is now reserved for select patients in whom these newer imaging modalities are unavailable.

### 3.3. Myocardial Perfusion Imaging (MPI)

Myocardial Perfusion Imaging (MPI) has an evolving role in cardio-oncology beyond traditional assessment of coronary artery disease (CAD). While MPI has been used to assess ischemia in patients with CAD, PET-based MPI can assess myocardial blood flow (MBF) and microvascular dysfunction associated with cancer therapies, in particular microvascular injury due to anti-VEGF agents or vasospasm related to 5-fluorouracil and its oral pro-drug capecitabine [17]. These changes may occur before left ventricular dysfunction and serve as markers of early cardiotoxicity.

While advanced imaging methods like PET and SPECT are critical for the noninvasive assessment of perfusion deficits in patients, their utility in oncology is also important for the long-term risk assessment of cancer survivors [18]. State-of-the-art quantitative PET MPI with the new F-18 flurpiridaz tracer can measure absolute myocardial blood flow and has demonstrated higher sensitivity for detecting microvascular impairment, especially after cancer therapy, in the absence of obstructive CAD [19]. This provides the opportunity for risk-based early interventions and personalized monitoring for patients at higher risk.

Although prior myocardial infarction or revascularization can complicate MPI interpretation, the integration of artificial intelligence enhances diagnostic precision by improving motion correction, noise reduction, and multimodal data synthesis—particularly valuable in patients with complex cardiovascular histories following cancer treatment [17]. As cancer survivorship increases, MPI, particularly PET-based approaches, is emerging as a useful tool for identifying persistent or latent vascular injury and may support preventive cardiovascular care.

### 3.4. Metabolic Imaging with PET

Positron emission tomography (PET) enables noninvasive assessment of myocardial metabolism using radiotracers, most commonly ^18F-fluorodeoxyglucose (FDG). While FDG-PET is primarily used in oncology to identify tumors and evaluate response to treatments, such as in breast and lung cancer, it may be used in assessing changes in systemic and cardiac metabolism as well [20,21,22,23].

In cardio-oncology, while PET imaging utilizes ionizing radiation, its primary clinical goal is the high sensitivity detection of metabolic and inflammatory markers of cardiotoxicity without directly inducing any toxic effects. For example, FDG uptake in the myocardium may represent inflammation due to cancer treatments, e.g., myocarditis from immune checkpoint inhibitors, or oxidative stress from anthracyclines. Myocardial FDG uptake, which is patchy or elevated, may be an early indicator of cardiotoxicity, before any changes in structure and/or function, and provides an opportunity for early intervention [20]. This provides the assessments of cancer and cardiac risk concomitantly and allows for an integrated assessment of the tumor and heart during and post cancer therapy.

Metabolic network analysis of the whole body demonstrates systemic dysregulation in cancer and inflammation (e.g., lung cancer and post-COVID-19), highlighting metabolic dysregulation as common to both [24]. Emerging techniques such as deuterium metabolic imaging (DMI) remain largely investigational but may provide additional insights into metabolic pathways [25].

## 4. The Synergistic Value of a Multimodality Imaging Approach

### 4.1. A Comparative Analysis of Imaging Modalities

Imaging modalities each have their own advantages depending on the specific clinical question posed. (Table 1) For the case of obstructive coronary artery disease (CAD), CMR has the highest sensitivity (0.88), making it the most definitive for excluding the diagnosis of significant CAD. Conversely, for the diagnosis of CAD, PET is better than SPECT, providing more true-positive (sensitivity = 0.85 for PET and = 0.69 for SPECT) and true-negative results (specificity = 0.89–0.92 for PET and =0.75–0.82 for SPECT) [26].

In the evaluation of chemotherapy-induced cardiomyopathy, the choice of imaging is again case-specific. For the initial evaluation of left ventricular function, echocardiography is, due to accessibility, often first-line. Yet, for a more precise and reproducible assessment of left ventricular ejection fraction (LVEF), CMR is considered the gold standard. This is because echocardiography can underestimate LVEF values [27]. For LVEF assessments, nuclear imaging (e.g., MUGA) is reproducible and thus is ideal for change monitoring throughout therapy, as it is crucial for long-term monitoring [28]. In clinical practice, imaging selection is guided by availability, patient risk, and clinical context. Echocardiography is typically used for routine surveillance, while CMR provides more precise evaluation when needed, and nuclear imaging may offer additional functional or mechanistic insights in selected patients.

In addition, computed tomography (CT), including coronary artery calcium scoring and CT angiography, plays an important role in detecting radiation-induced coronary artery disease and accelerated atherosclerosis in cancer survivors.

**Table 1:** Comparative table showing the diagnostic performance and best use of different imaging modalities.

Imaging Modality	Sensitivity	Specificity	Best Use	Advantages	**Limitations**
MUGA (Radionuclide Angiography) [20].	95–98% for LVEF.	90–95% for LVEF.	Serial LVEF monitoring during chemotherapy.	Highly reproducible LVEF measurements Minimal inter-observer variability Established guideline standard No geometric assumptions.	Radiation exposure Limited functional/ structural data No tissue characterization Poor temporal resolution.
2D Echocardiography [29].	70–85% for LVEF.	75–85% for LVEF.	First-line screening and monitoring.	Widely available No radiation Real-time imaging Cost-effective Portable.	Operator-dependent Limited acoustic windows Geometric assumptions High inter-observer variability.
3D Echocardiography [29].	85–92% for LVEF.	88–94% for LVEF.	Improved volumetric assessment.	More accurate than 2D Reduced geometric assumptions Better reproducibility.	Requires specialized equipment Lower temporal resolution Image quality dependent Limited availability.
GLS (Global Longitudinal Strain) [30].	80–90% for early dysfunction.	85–92% for early dysfunction.	Early subclinical cardiotoxicity detection.	Detects subtle dysfunction Predicts LVEF decline Earlier than conventional LVEF.	No standardized cutoff values Vendor variability Requires specific software Load-dependent.
CMR (Cardiac MRI) [31].	95–99% for LVEF. 90–95% for fibrosis.	95–98% for LVEF. 92–96% for fibrosis.	Gold standard for volumes, tissue characterization.	Highest accuracy/reproducibility Tissue characterization (LGE, T1/T2 mapping) No radiation Comprehensive assessment.	Expensive Limited availability Long acquisition time Contraindications (devices, claustrophobia).
SPECT (Single-Photon Emission CT) [32].	85–90% for perfusion defects.	80–88% for perfusion defects.	Myocardial perfusion assessment.	Established perfusion protocols Widely available Combined perfusion + function Prognostic data.	Radiation exposure Lower resolution than PET Attenuation artifacts Limited metabolic data.
PET (Positron Emission Tomography) [33].	90–95% for perfusion 85–92% for metabolism.	88–94% for perfusion 90–95% for inflammation.	Early metabolic dysfunction, inflammation detection.	Superior spatial resolution Quantitative blood flow Metabolic/inflammatory imaging Early injury detection.	High cost Limited availability Short-lived tracers Radiation exposure Requires cyclotron (some tracers).
PET/CT Hybrid [34].	92–96% (combined).	90–95% (combined).	Simultaneous metabolic + anatomical assessment.	Precise anatomical localization Combined functional/structural data Attenuation correction Coronary calcium scoring.	Highest radiation dose Very high cost Limited availability Complex protocols.
PET/MRI Hybrid [35].	93–97% (combined).	92–96% (combined).	Comprehensive metabolic + tissue characterization.	No radiation from MRI component Superior soft tissue contrast Simultaneous metabolic + tissue data Ideal for research.	Extremely limited availability Highest cost Longest scan time Technical complexity.

### 4.2. The Power of Hybrid Imaging

Regarding assessing cancer in the heart, hybrid imaging techniques that merge PET, CT, MRI, and SPECT deliver top-notch structural and functional information all in a single examination. In the rapidly growing field of cardio-oncology, this combination is important, giving doctors the chance to see both the cancer and the health of the heart at the same time [36]. With a heightened need for more accurate and specific diagnoses and treatments for cardiac diseases, the integration of these imaging techniques plays an essential role in the process [37].

PET/CT is regarded as a standard tool in oncology. In cardio-oncology, simultaneous evaluation of tumor response and potential cardiac complication side effects is possible. One example is a therapy assessment on a primary tumor and screening for perfusion defect due to chemotherapy-induced ischemia. PET/MRI is promising for clinical purposeful integrated oncology because of the metabolic signal, e.g., FDG uptake for inflammation, and the more sophisticated cardiac body architecture function. This represents a complete assessment of oncology and cardio toxicity [38]. Both modalities are appreciated in the holistic management of oncology patients [39].

**Figure 2:**
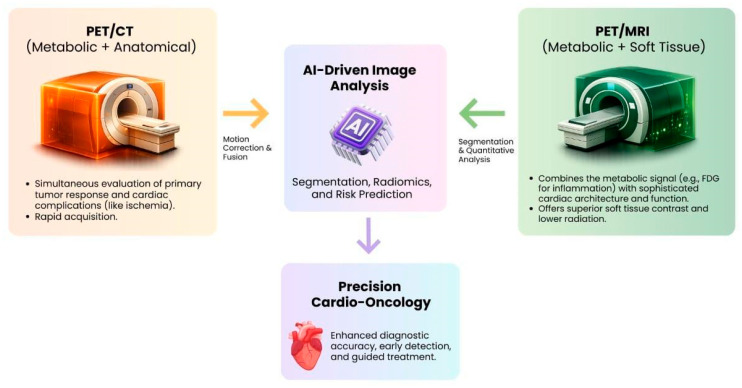
Graphically depicts the hybrid imaging modalities and their AI integration in Cardio-oncology. Abbreviations: AI: Artificial Intelligence, CT: Computed tomography, FDG: Fluorodeoxyglucose, MRI: Magnetic Resonance Imaging, PET: Positron Emission Tomography.

The rich and intricate data sets from these hybrid studies are almost impossible to analyze without employing artificial intelligence image analysis and interpretation. This creates its own issues in the realm of data management and quality control [39] (Figure 2).

### 4.3. Imaging Across the Cardio-Oncology Care Continuum

The role of imaging in cardio-oncology spans the entire continuum of care, including baseline assessment, monitoring during therapy, and long-term survivorship evaluation. At baseline, echocardiography with assessment of left ventricular ejection fraction (LVEF) and global longitudinal strain (GLS) is typically used for cardiovascular risk stratification prior to initiating cancer therapy. In selected high-risk patients, additional imaging modalities may be considered to further characterize myocardial structure or function. During active cancer therapy, serial echocardiography remains the primary modality for monitoring cardiac function. However, nuclear imaging techniques, particularly PET-based approaches, may identify early metabolic or microvascular alterations before overt functional decline becomes apparent. In the post-therapy and survivorship phase, imaging is essential for detecting late cardiovascular complications, including microvascular dysfunction, persistent inflammation, and radiation-induced coronary artery disease. Modalities such as PET myocardial perfusion imaging and CT-based coronary assessment may provide valuable information for long-term risk stratification and management. This framework highlights the complementary role of different imaging modalities in supporting a more comprehensive and individualized approach to cardio-oncology care.

## 5. Molecular Imaging: Visualizing Pathophysiology at a Cellular Level

### 5.1. The Paradigm Shift from Function to Pathology

In the past 20 years, the imaging of the cardiovascular system has moved from assessments of gross cardiac function to mechanisms that occur before the establishment of irreversible changes [40]. While Echo, MRI, and CT assess ventricular volume and fibrosis, these parameters are changes from late-stage disease. injuries. In cardio-oncology, this shift is particularly important since the aim is to assess for any cardiotoxicity before there is a drop in the LVEF so that a therapeutic intervention can be applied during active cancer treatment [41].

On the other hand, molecular imaging techniques such as PET and SPECT that show the physician pathophysiological changes at the cell-subcellular levels, such as metabolic dysregulation, inflammatory cell infiltration, fibroblast activation, and apoptotic cell signaling, are changes that occur before dysfunction and present an opportunity for intervention and a potential alteration in the disease continuum [42].

For instance, in inflammatory cardiomyopathies such as cardiac sarcoidosis, FDG-PET facilitates the visualization of inflammation even in patients with preserved ejection fraction, thereby guiding the commencement of immunosuppressive therapy. This idea can be used directly in cancer treatment, where drugs like immune checkpoint inhibitors can cause similar inflammatory cardiomyopathies. Furthermore, FDG uptake patterns correlate with adverse cardiac outcomes, underlining their prognostic relevance [42,43]. This represents a significant advancement, suggesting a potential role for imaging as a biomarker of disease activity and therapeutic response. However, it also creates challenges, such as variability in protocols and the need to harmonize these molecular approaches to ensure consistency in a diverse patient population.

### 5.2. Targeted Molecular Pathways and Novel Radiotracers

FDG is the most well-established tool for examining myocardial metabolism. Usually, the heart mainly prefers oxidizing fatty acids. However, in pathological conditions, the heart shifts towards glycolytic metabolism. This metabolic shift is a hallmark of chemotherapy-induced cardiotoxicity, especially anthracyclines. Therefore, FDG-PET imaging can be used to assess for early metabolic changes/injury. FDG utilizes the metabolic shift to diagnose dysfunctional but still viable myocardium. Some strategies to improve the specificity of the studies by reducing normal myocardial glucose uptake include prolonged fasting and the use of a diet high in fats [44].

Cardiac FAPI uptake has been studied in areas of active fibrinogenesis post-myocardial infarction, as well as in hypertrophic cardiomyopathy [30]. Increased FAPI uptake has been associated with adverse cardiac remodeling and may have prognostic implications, including risk stratification for sudden cardiac death. In the context of cardio-oncology, FAPI imaging targets activated fibroblasts and may allow for detection of early fibrotic remodeling associated with cardiotoxic therapies such as anthracyclines and HER2-targeted agents, potentially identifying changes before established scar formation on cardiac MRI [31]. However, studies about FAPI in cardiology are still limited. Clinical applicability varies depending on tracer validation and availability. Apoptosis, the process of programmed cell death, leads to the progressive loss of cardiomyocytes during ischemia-reperfusion injury and chemotherapy-related heart damage. Radiolabeled annexin V probes target the phosphatidylserine on the outer leaflets of the membrane of apoptotic cells. While there is evidence of annexin V uptake in myocardial infarctions, there is too much non-specific uptake, too fast a clearance, and too much radiation exposure to warrant use in a clinical setting [32].

MIBG (Iodine-123 metaiodobenzylguanidine) SPECT is a validated prognostic tool in heart failure, where decreased uptake is associated with decreased survival and increased risk of sudden death and ventricular tachycardia. PET tracers have better resolution than SPECT, and are being developed, like ^11^C-hydroxyephedrine (HED) and newer ^18^F-Labeled tracers, including ^18^F-LMI1195. These tracers have been shown to evaluate heart failure and chemotherapy-induced cardiotoxicity. However, do not have clinical availability/or have strict regulations associated with them [33].

Collectively, these tracers have moved away from basic structural and functional measurements to more sophisticated biological measurements. (Table 2) While it is considered the gold standard for onco- cardiology, it still has limitations and serves to drive the development of more advanced tools like FAPI, which target advanced monitoring to the next level. While FDG VISF scans have shown some evidence of heart failure, their use has been limited as a result of the stringent regulations and availability of the tracers [34].

**Table 2:** Summarizes the key molecular radio tracers in Cardio-oncology.

Tracer	Mechanism	Primary Clinical Application	Key Limitation
^18^F-FDG Fluorine-18 Fluorodeoxyglucose [35].	Glucose metabolism A glucose analog that accumulates in metabolically active tissues, reflecting cellular energy consumption.	Viable myocardium assessment, myocardial inflammation detection, and cardiac sarcoidosis.	Requires 12–18 h fasting and a low-carb diet preparation; physiologic myocardial uptake can reduce specificity; limited spatial resolution.
FAPI Fibroblast Activation Protein Inhibitor [45].	Fibroblast activation protein (FAP) Binds specifically to activated fibroblasts expressing FAP, marking areas of active fibrosis and tissue remodeling.	Post-MI cardiac remodeling, myocardial fibrosis assessment, and diastolic dysfunction evaluation.	Limited clinical data and validation studies; not yet FDA-approved for cardiac use; standardization of imaging protocols needed.
Annexin V Annexin V (^123^I or ^99m^Tc labeled) [44].	Phosphatidylserine (apoptosis marker) Binds to phosphatidylserine exposed on the outer membrane of apoptotic cells, detecting programmed cell death.	Early cardiotoxicity detection, apoptosis imaging in anthracycline therapy, and acute cardiac rejection monitoring.	Blood pool clearance issues affecting image quality; radiation burden concerns for repeated imaging; limited commercial availability.
^123^I-MIBG Iodine-123 meta-Iodobenzylguanidine [46].	Cardiac sympathetic nervous system Norepinephrine analog is taken up by presynaptic sympathetic nerve terminals, reflecting cardiac autonomic innervation.	Heart failure prognosis, arrhythmia risk stratification, and cardiotoxicity-induced autonomic dysfunction.	Limited availability in many centers; requires specialized imaging protocols; expensive; multiple medications interfere with uptake.
^68^Ga-DOTATATE Gallium-68 DOTA-octreotate [47].	Somatostatin receptors (SSTR) Binds to somatostatin receptors overexpressed on inflammatory cells and in cardiac masses.	Cardiac tumor detection, inflammatory cardiomyopathies, and neuroendocrine tumor cardiac metastases.	Limited cardiac-specific data; primarily used for oncologic indications; not specific for cardiotoxicity.
^15^O-H_2_O Oxygen-15 Water [48].	Myocardial perfusion A freely diffusible tracer that measures absolute myocardial blood flow and coronary flow reserve.	Coronary microvascular dysfunction, chemotherapy-induced vascular toxicity, and absolute blood flow quantification.	Very short half-life requires an on-site cyclotron; expensive; complex quantification protocol.

## 6. Innovations on the Horizon: Artificial Intelligence and New Technologies

### 6.1. Artificial Intelligence (AI) and Radiomics: The Democratization of Expertise

Artificial intelligence (AI) and advanced computational methods are increasingly being applied in cardiovascular imaging by analyzing large amounts of information, including tissue texture, patterns, and function, from various imaging modalities (e.g., PET, CT, and MRI). In cardio-oncology, these tools are being actively investigated for potential clinical application, particularly in the early prediction of cancer therapy-related cardiac dysfunction (CTRCD) [49].

One of the most significant changes brought about by AI is the equalization of expertise levels. As an example, automated segmentation algorithms automated segmentation of the left ventricle reduces inter-observer variability in LVEF assessment, controlling the left ventricle side of the gaps, which minimizes the variability between observers in measuring the LVEF. Small changes which may improve the detection of subtle changes in cardiac function during chemotherapy monitoring are real changes and not measurement error [50].

AI-based methods may improve motion correction and noise reduction in cardiac PET imaging. This has led to shorter imaging acquisitions with less radiation, improved image quality, and decreased patient dose [51]. Some radiomics models (high throughput extraction of quantitative features including shape, intensity and texture of medical images like CT, MRI and PET) are beginning to successfully predict and differentiate non-ischemic types of diffuse myocardial fibrosis and edema from anthracycline and radiation therapy, and those induced by ischemic insults [52].

**Figure 3:**
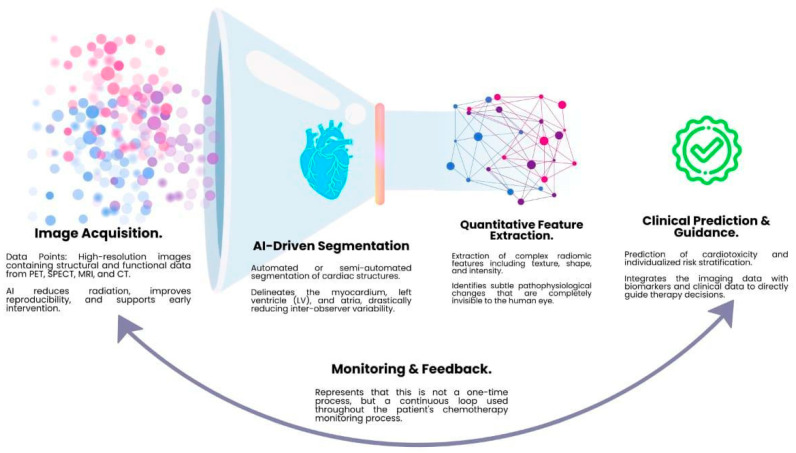
Showing the AI and radiomics workflow for cardio oncology imaging.

Despite this, the potential of AI is to support, and not to substitute, clinical judgment, and future advances are likely to rely on multifacility validation and “explainable AI” methods. (Figure 3) Clinicians need to understand the features that are most relevant to an algorithm’s prediction of a given toxicity to make reasoned decisions about whether to pause critical treatment for patients with cancer. The trust that can be placed in these models is limited by the “black box” characteristic of the present models, and differences in imaging protocols continue to be a barrier to the broad reproducibility [53]. An overview of AI and radiomics integration in imaging analysis is shown in Figure 3.

### 6.2. Advances in Hardware and Camera Technology

The cutting edge of hardware technology has expanded new possibilities in cardiovascular imaging. Total body PET scanners with new axially extended field of view detectors can simultaneously perform scans of cardiac function and assess the burden of metastatic tumors while with the potential to reduce scan time and radiation exposure. This is particularly important for cancer survivors and patients who need to undergo longitudinal scans frequently to monitor the progression of their disease [29].

The latest advances in digital photon counting technology and new detector materials have significantly improved the speed and quality of data collected in any given time span. These advancements assist in providing virtually undetectable earlier stages of disease processes, such as, but not limited to, early inflammation due to immune checkpoint inhibitors and microvascular dysfunction, among many others [30].

Despite their newness, hybrid imaging devices such as PET/MR systems combine the molecular details of imaging with the structural information obtained via superior soft tissue contrast. This has already shown promise in the field of pediatric oncology and in young survivors. It offers a radiation-free means of assessing structures while characterizing inflammation and fibrosis in the myocardium [31]. All the while, newer SPECT hardware, such as solid-state cadmium zinc telluride (CZT) detectors, has made the lower doses of tracers needed to assess for chemo-induced vascular toxicity with the newer capabilities of dynamic acquisition and quantification of myocardial blood flow.

## 7. Clinical and Regulatory Barriers to Implementation and Dissemination

### 7.1. The Critical Lack of Consensus and Standardized Protocol

There are implementation challenges regarding the uneven availability of cardio-oncology imaging methods, with the low-and middle-income countries (LMICs) prevailing as the most negatively affected [54]. Most LMICs’ health system structures are reliant upon baseline echocardiography (without GLS, PET, or radiochemistry access)—meaning that subtle chemotherapy-induced microvascular injuries or early CTRCD go undetected until LVEF decreases significantly [55]. A global deficit of cardio-oncology-trained medical professionals also hinders further scaling. Even in upper-income countries, an estimated 60% of cardiologists do not have training on interpreting molecular images. This lack of training leads to a loss in utilization of PET/CMR in non-specialist centers. More localized factors, such as the differing capabilities in tracer preparation from center to center, also contribute to the inconsistent use of fully validated molecular tools for CTRCD monitoring [56].

### 7.2. Economic and Regulatory Challenges

Novel radiopharmaceuticals (e.g., FAPI tracers) face the lengthy approval process due to the stringent regulatory approvals and the stringent biomarker qualification that prioritizes cancer tumors over cardiac toxicity. There is a mismatch with cardio-oncology having a dual focus on tumor response and cardiovascular safety. Regulatory approvals for AI imaging tools (e.g., automated LVEF segmentation) also face similar challenges [57]. In the EU and the U.S., data protection laws slow down the dissemination of multicenter data for validation. Clinicians are reluctant to use tools for routine CTRCD monitoring due to ambiguous regulatory policies on ‘cardio-oncology-specific AI.’ Even tools that are evidence-based can take 5 to 7 years to transition from trial to clinic due to these bottlenecks [54].

In cardio-oncology, molecular imaging lacks coverage from payers due to uncertainty around cost-effectiveness. For example, even though PET-MRI lowers the amount of radiation directed to pediatric cancer survivors, U.S. private insurers do not offer coverage for CTRCD due to the absence of data concerning cost and outcomes in the long term. Even more so in LMICS, the systemic undercapacity in radiology staff training and scanner maintenance means that even PET scanners that are donated remain unused [58,59]. As cancer survivorship continues to rise, the lack of funding for cardio-oncology systems will continue to exacerbate the inequities [60].

### 7.3. Radiation Exposure and Benefit–Risk Considerations

Cancer patients are frequently exposed to cumulative ionizing radiation from diagnostic imaging and radiotherapy, raising important considerations when incorporating nuclear imaging into routine care. Modalities such as PET/CT contribute additional radiation exposure, which is particularly relevant in younger patients and long-term cancer survivors. This concern is amplified in pediatric populations, where lifetime radiation risk is higher.

Strategies to mitigate exposure include the use of low-dose imaging protocols, stress-only imaging approaches, and the increasing adoption of hybrid modalities such as PET/MRI, which reduce radiation burden while preserving diagnostic capability. Consequently, the use of nuclear imaging in cardio-oncology should be guided by careful benefit–risk assessment, ensuring that incremental diagnostic value justifies additional radiation exposure.

## 8. Challenges and Limitations in Literature

### 8.1. Clinical Protocol Heterogeneity and Evidence Gaps

Reliance on particular methodologies is damaging to the clinical confidence in cardio-oncology imaging. Data for GLS cutoffs for CTRCD varies as much as 15–20% for differing studies, and there is no industry standard to measure strain. This may lead to decisions that are in direct conflict with one another [35]. For example, a general cardiologist in a non-expert center may clear a patient for continuation of the anthracycline based on a local GLS cutoff, but the cardio-oncology expert will hold therapy with respect to the patient based on a completely different threshold. The voids of gap reproducibility are also evident in the area of molecular imaging. In the case of microvascular injury, the inability to make comparisons among the trial data due to inconsistent tracer dosing for FDG-PET leaves the clinician with the absence of a consistently applicable frame of reference for early toxicity quantification [35,36]. This review primarily focuses on adult populations, and the applicability of these findings to pediatric cardio-oncology requires further dedicated investigation.

### 8.2. Implementation Barriers: Economic Constraints, Workforce Gaps, and Regulatory Challenges

**Figure 4:**
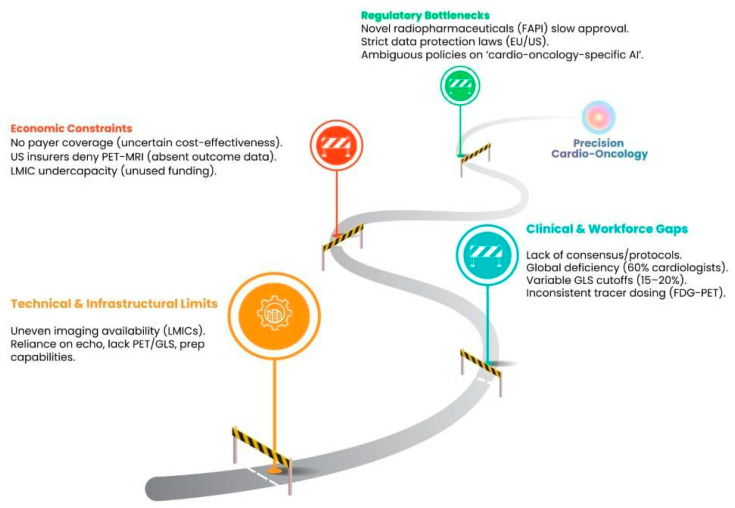
Limitations and barriers to implement advanced cardio-oncology imaging.

The absence of a standard set of clearly defined and lexically consistent cardio-oncology imaging surrogate endpoints creates obstacles for updating the guidelines and conducting meta-analyses [38]. As an example, 40% of the CTRCD studies in the recent past state “LVEF decline ≥ 10%” as the primary endpoint, and the other 40% state “GLS decline ≥ 15%”; however, there is no consistent endpoint to capture the cardiac injury as a result of the therapies. This situation is compounded by the dearth of multicenter studies that include diverse populations, such as those from LMICs, older survivors, and those who are on combination immunotherapy and chemotherapy. In the absence of these studies, there is a reliance on expert opinions to form these guidelines, and this is insufficient, as the information does not apply to a general population [38,39] (Figure 4).

### 8.3. Limitations of the Narrative Review

As a narrative review, the study selection and synthesis may be subject to search and selection bias. This review did not utilize a formal systematic met- analysis framework or risk of bias assessment for every included study. While providing a broad overview of cardio-oncology imaging and identifying the gaps in this field, being a narrative review it lacks in the quantitative aspect of the analysis.

## 9. Future Directions and Research Priorities in Precision Cardio-Oncology

The expansion of cardio-oncology has been matched by stunning progress in multimodality imaging and advanced analytics. Echocardiography, and specifically strain imaging, remains the practical frontline modality [7,48]. CMR and PET increasingly offer first-grade diagnostic and prognostic information [49,61]. However, throughout the literature, there is a unifying theme: the greatest gap is poor-quality evidence. All the recommendations are based on small or heterogeneous populations, typically limited to single-center data [50].

In the future, a number of priorities are apparent. First, there is a need for future prospective multicenter trials to prove imaging biomarkers and make firm connections between imaging results and long-term outcomes. These trials should compare not only traditional modalities but also AI-based workflows and hybrid imaging approaches [52]. Second, registries and multicenter networks need to systematically gather real-world cardiotoxicity data. These efforts would enable guideline developers to transition from expert consensus to evidence-based guidelines [12]. In parallel, the establishment of large-scale registries and collaborative cardio-oncology networks will be essential to generate real-world evidence and improve external validity. Standardization of imaging protocols, including tracer selection, acquisition parameters, and reporting frameworks, is critical to improve reproducibility and enable meaningful comparisons across studies. Furthermore, dedicated training programs in cardio-oncology imaging are needed to address workforce gaps and ensure appropriate interpretation and clinical integration of advanced imaging modalities.

Third, the convergence of imaging with genetics, circulating biomarkers, and digital health platforms holds out the promise of an age of precision cardio-oncology. Deep phenotyping, when combined with cutting-edge imaging, might make individualized risk stratification and truly personalized treatment regimens possible [12,53]. Lastly, international cooperation is needed. Only with global harmonization can the practice move from disparate approaches to standardized, validated, and fair care.

## 10. Conclusions

Precision cardio-oncology has become vital as cancer survivorship increases and cardiovascular complications progressively affect long-term outcomes. This review shows that nuclear imaging represents an emerging complementary tool that can support the transition from functional surveillance toward a more mechanism-driven, precision-based approach. Nuclear techniques may enable earlier detection of cardiotoxic injury before the development of overt structural or functional decline by allowing us to observe myocardial perfusion, metabolism, inflammation, and microvascular integrity. Quantitative PET myocardial perfusion imaging enables the early detection of microvascular dysfunction linked to antiangiogenic agents and fluoropyrimidines. Meanwhile, metabolic and inflammatory imaging, especially with ^18F-FDG, has proven effective in identifying immune checkpoint inhibitor-associated myocarditis and therapy-related myocardial inflammation. Hybrid platforms like PET/CT and PET/MRI make precision care even better by combining cancer testing with tests for cardiovascular disease at the same time. This helps with personalized monitoring and management plans.

Nuclear imaging provides a critical molecular bridge that allows for identifying subclinical metabolic and inflammatory changes before irreversible structural damage occurs by using advanced imaging techniques enhanced by AI driven radiomics. In general, nuclear imaging is likely to play an increasingly important complementary role in precision cardio-oncology. It is a powerful link between molecular pathophysiology and clinical decision-making that can help improve cardiovascular outcomes throughout the cancer care continuum.

## Data Availability

No new data were created or analyzed in this study. Data sharing is not applicable to this article.
